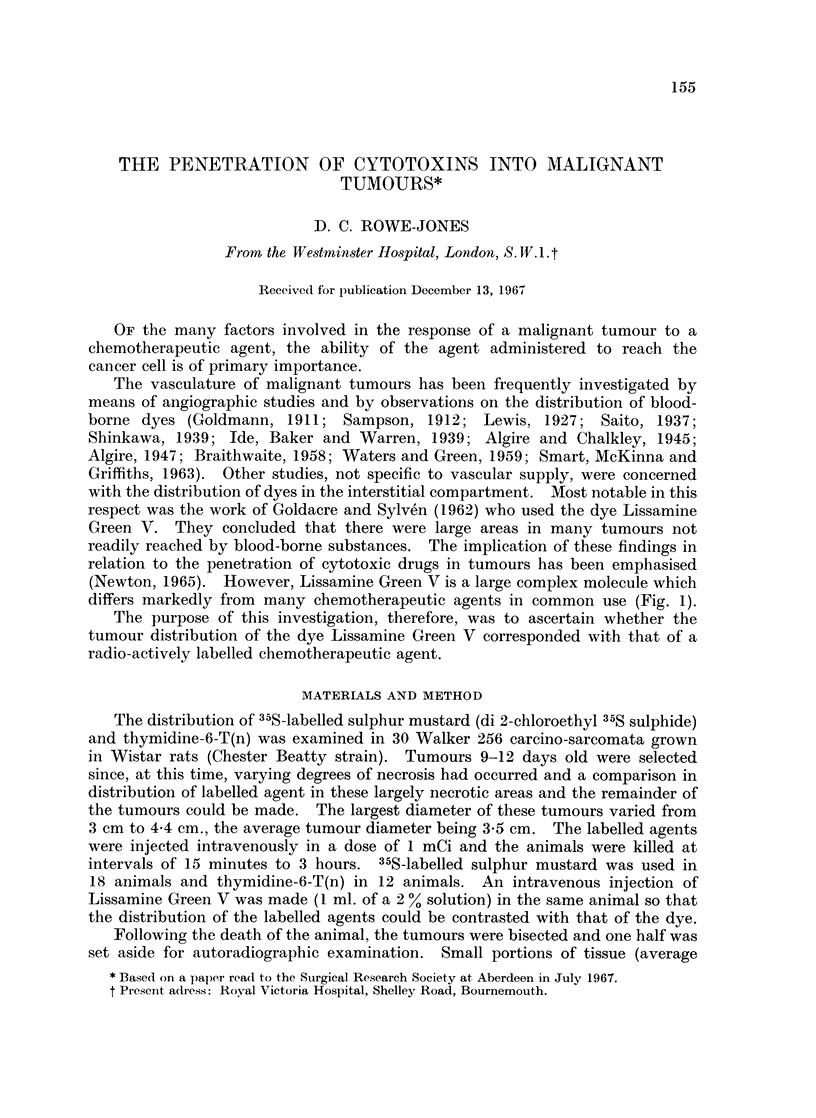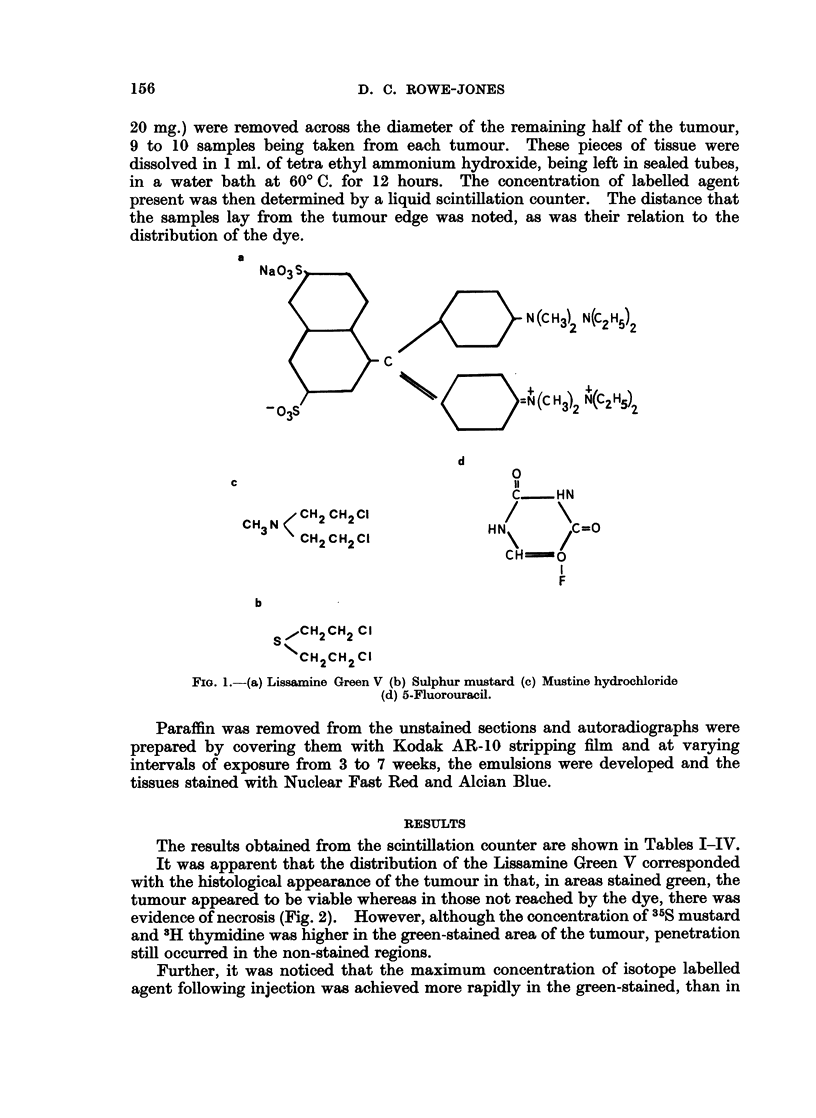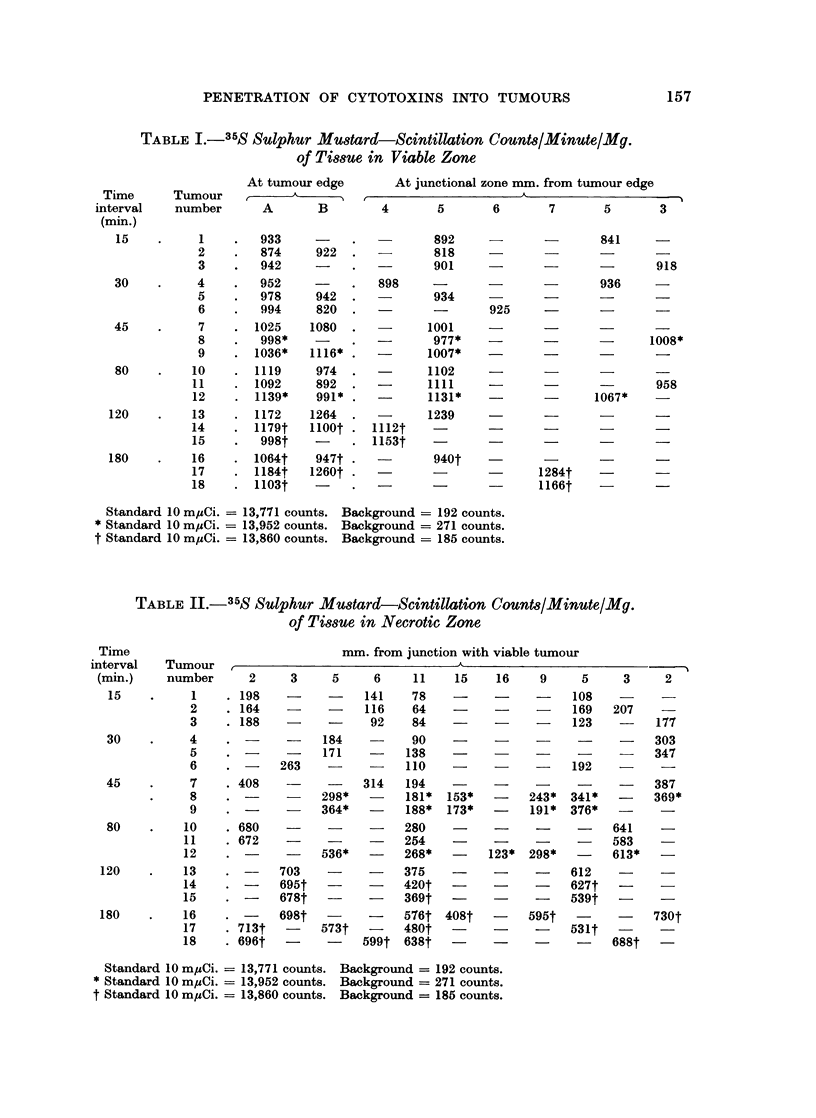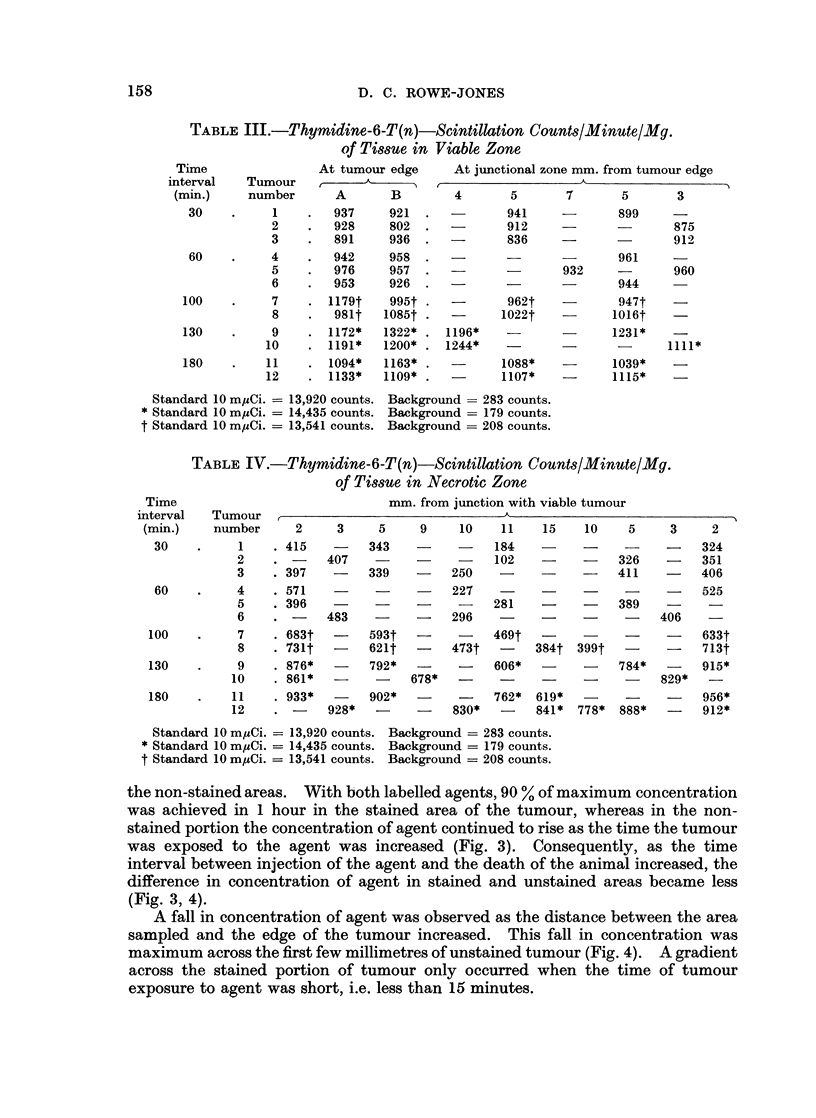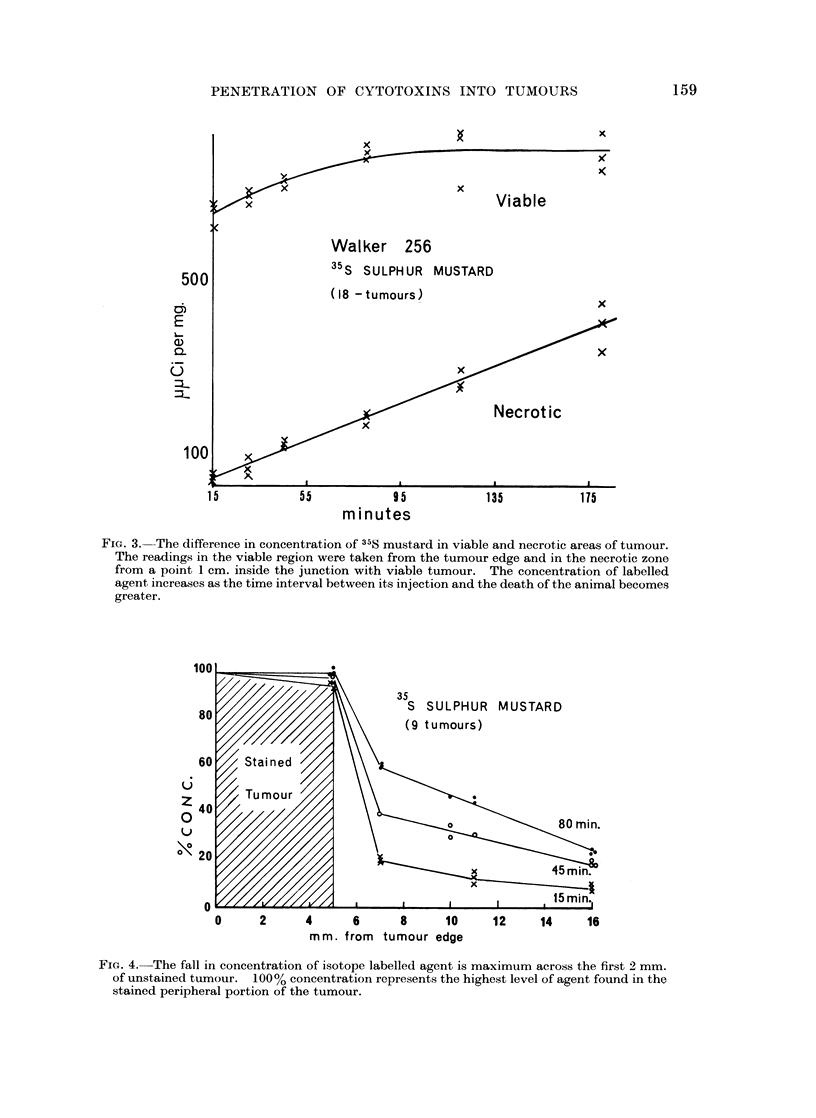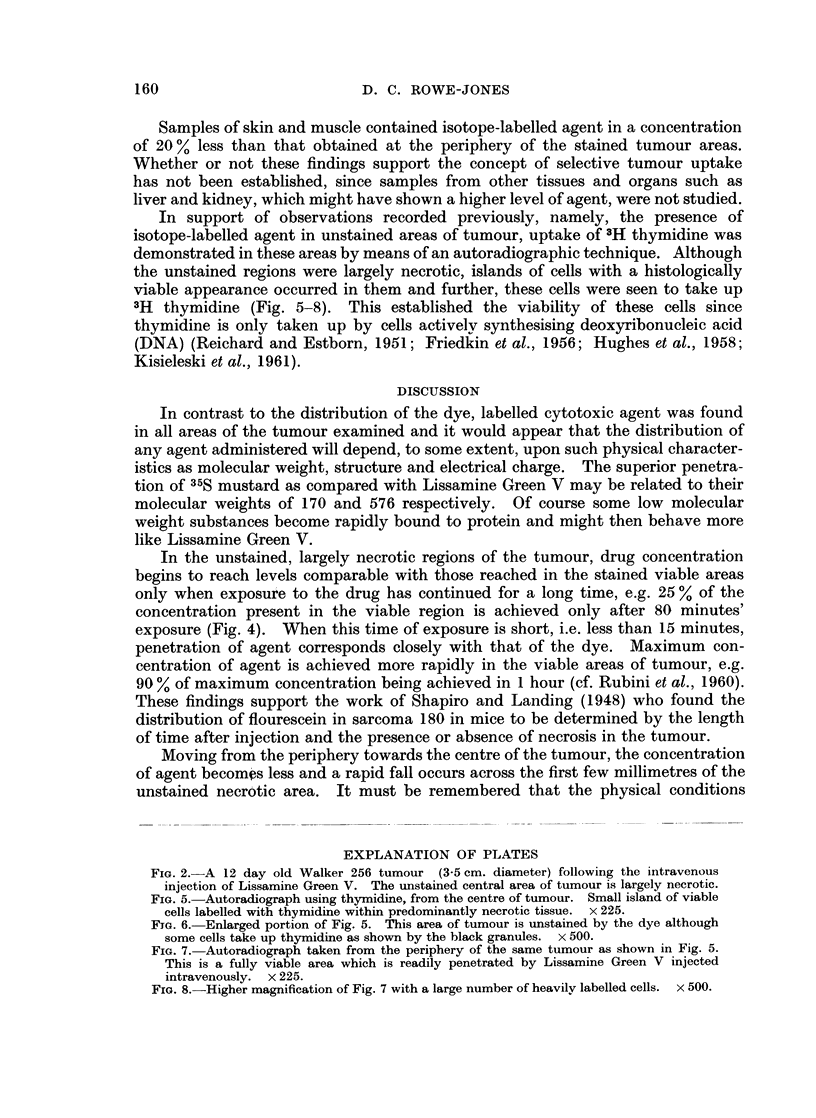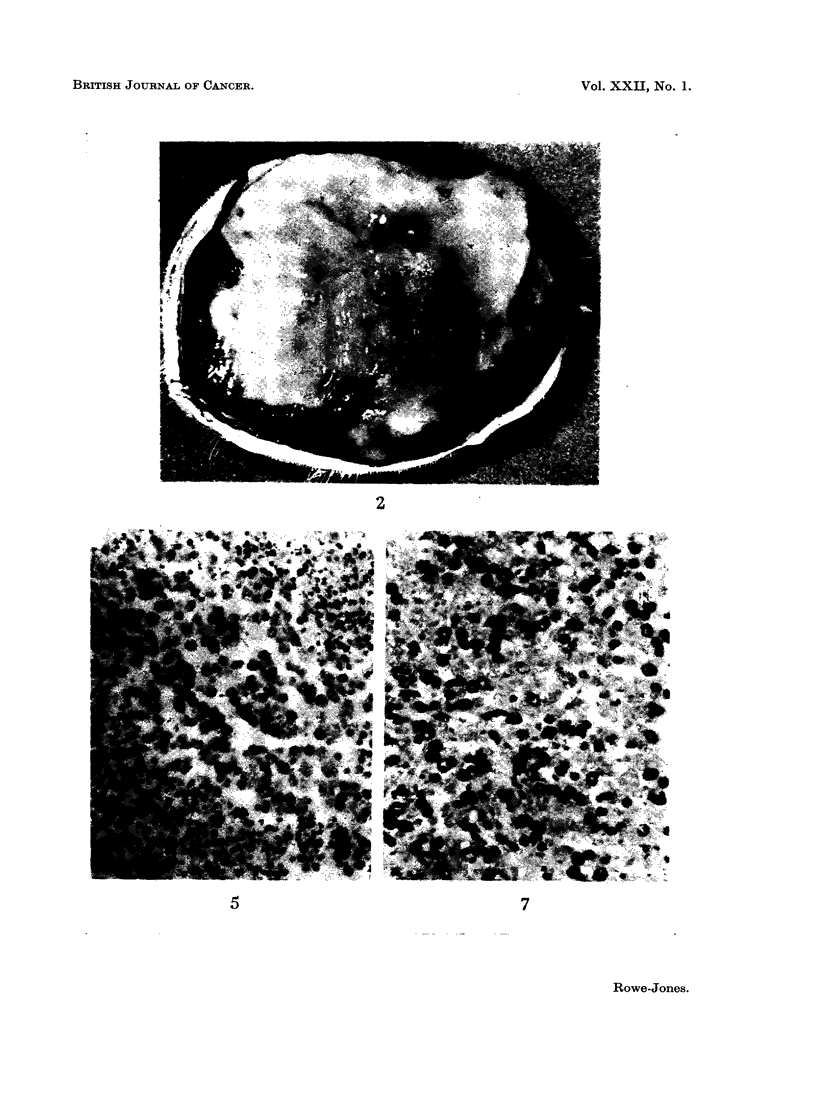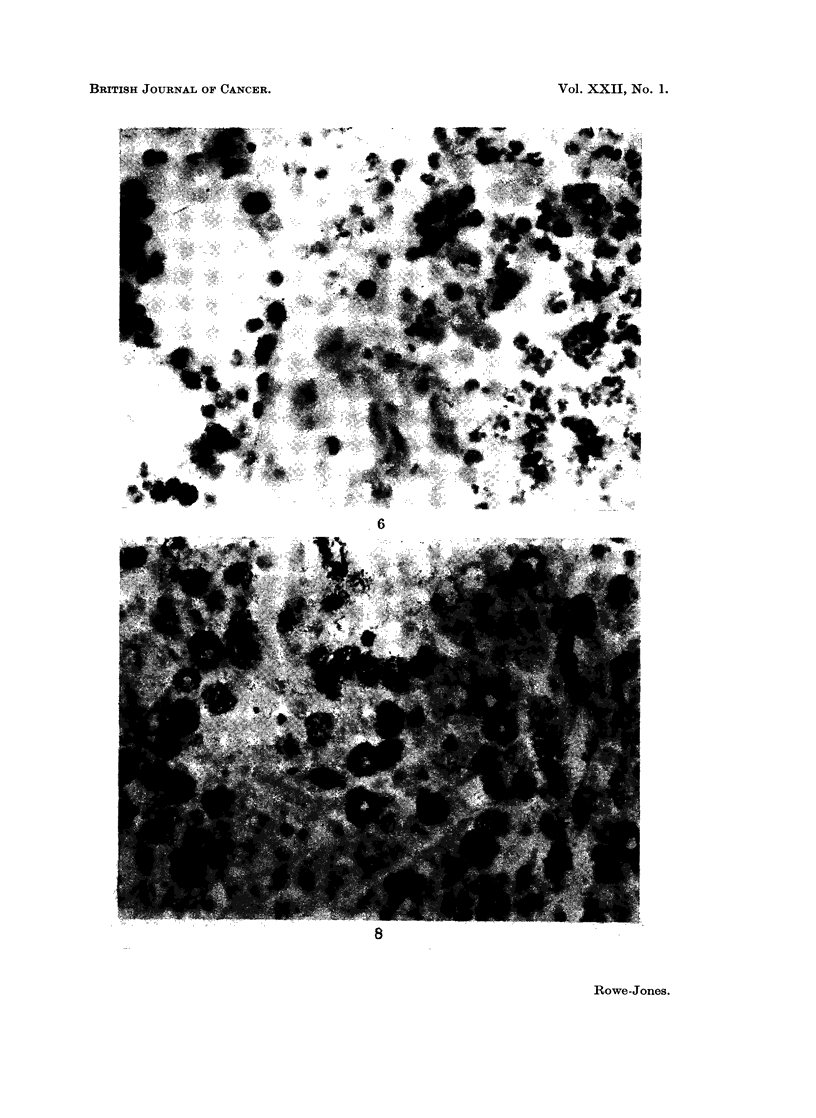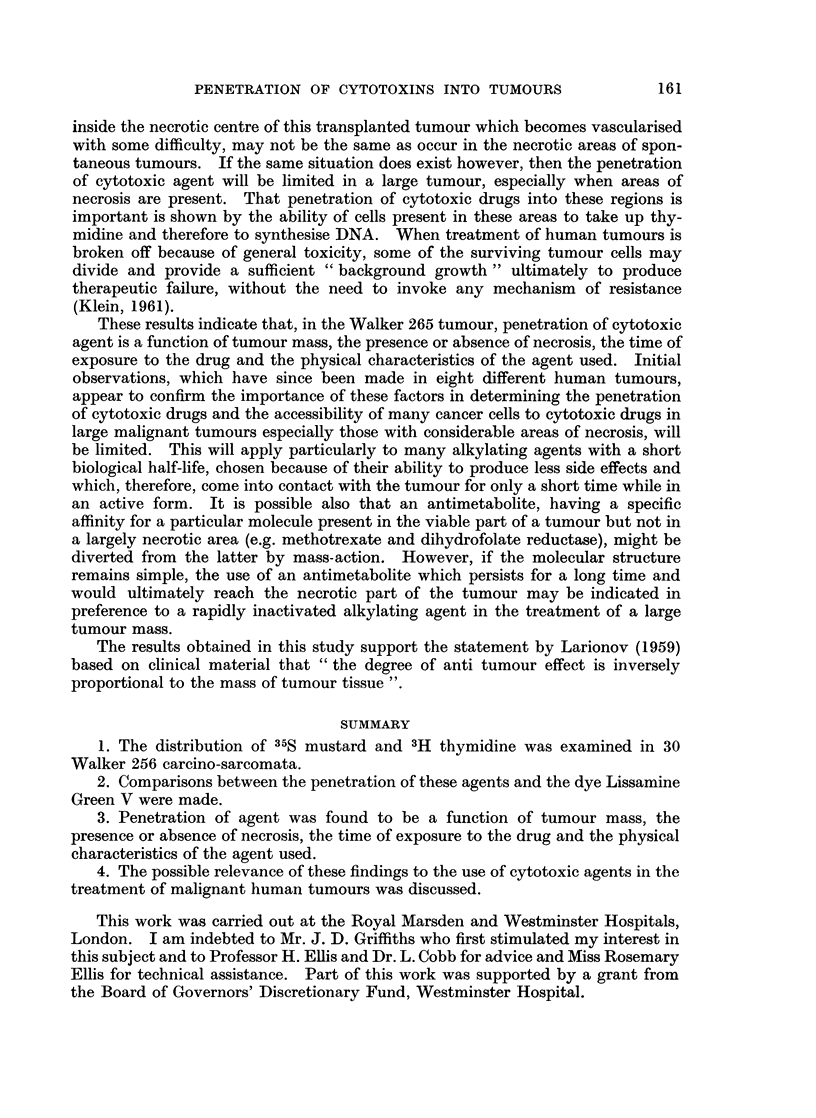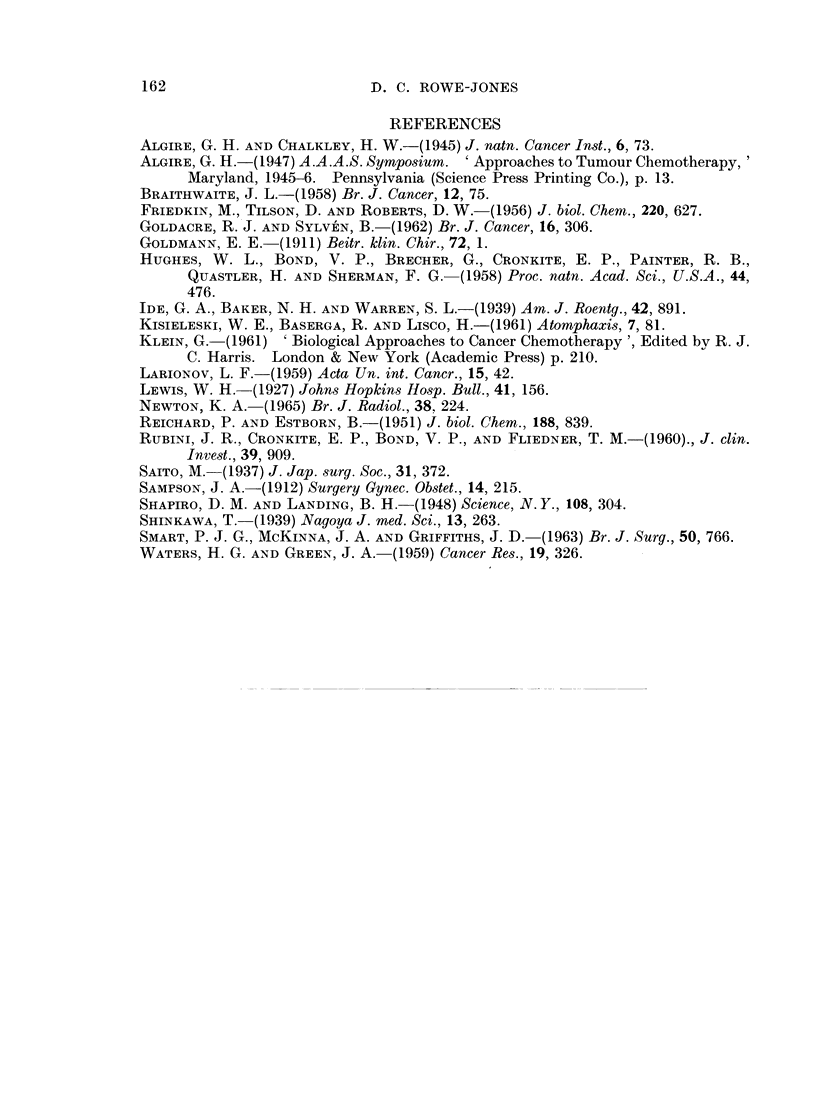# The penetration of cytotoxins into malignant tumours.

**DOI:** 10.1038/bjc.1968.22

**Published:** 1968-03

**Authors:** D. C. Rowe-Jones

## Abstract

**Images:**


### THE PENETRATION OF CYTOTOXINS INTO MALIGNANT TUMOURS*

D. C. ROWE-JONES

*From the Westminster Hospital, London, S.W.1.*†

Received for publication December 13, 1967

OF the many factors involved in the response of a malignant tumour to a chemotherapeutic agent, the ability of the agent administered to reach the cancer cell is of primary importance.

The vasculature of malignant tumours has been frequently investigated by means of angiographic studies and by observations on the distribution of blood-borne dyes (Goldmann, 1911; Sampson, 1912; Lewis, 1927; Saito, 1937; Shinkawa, 1939; Ide, Baker and Warren, 1939; Algire and Chalkley, 1945; Algire, 1947; Braithwaite, 1958; Waters and Green, 1959; Smart, McKinna and Griffiths, 1963). Other studies, not specific to vascular supply, were concerned with the distribution of dyes in the interstitial compartment. Most notable in this respect was the work of Goldacre and Sylvén (1962) who used the dye Lissamine Green V. They concluded that there were large areas in many tumours not readily reached by blood-borne substances. The implication of these findings in relation to the penetration of cytotoxic drugs in tumours has been emphasised (Newton, 1965). However, Lissamine Green V is a large complex molecule which differs markedly from many chemotherapeutic agents in common use (Fig. 1).

The purpose of this investigation, therefore, was to ascertain whether the tumour distribution of the dye Lissamine Green V corresponded with that of a radio-actively labelled chemotherapeutic agent.

### MATERIALS AND METHOD

The distribution of $^{35}$S-labelled sulphur mustard (di 2-chloroethyl $^{35}$S sulphide) and thymidine-6-T(n) was examined in 30 Walker 256 carcino-sarcomata grown in Wistar rats (Chester Beatty strain). Tumours 9–12 days old were selected since, at this time, varying degrees of necrosis had occurred and a comparison in distribution of labelled agent in these largely necrotic areas and the remainder of the tumours could be made. The largest diameter of these tumours varied from 3 cm to 4·4 cm., the average tumour diameter being 3·5 cm. The labelled agents were injected intravenously in a dose of 1 mCi and the animals were killed at intervals of 15 minutes to 3 hours. $^{35}$S-labelled sulphur mustard was used in 18 animals and thymidine-6-T(n) in 12 animals. An intravenous injection of Lissamine Green V was made (1 ml. of a 2 % solution) in the same animal so that the distribution of the labelled agents could be contrasted with that of the dye.

Following the death of the animal, the tumours were bisected and one half was set aside for autoradiographic examination. Small portions of tissue (average

* Based on a paper read to the Surgical Research Society at Aberdeen in July 1967.

† Present adress: Royal Victoria Hospital, Shelley Road, Bournemouth.

20 mg.) were removed across the diameter of the remaining half of the tumour, 9 to 10 samples being taken from each tumour. These pieces of tissue were dissolved in 1 ml. of tetra ethyl ammonium hydroxide, being left in sealed tubes, in a water bath at 60° C. for 12 hours. The concentration of labelled agent present was then determined by a liquid scintillation counter. The distance that the samples lay from the tumour edge was noted, as was their relation to the distribution of the dye.

FIG. 1.—(a) Lissamine Green V (b) Sulphur mustard (c) Mustine hydrochloride (d) 5-Fluorouracil.

Paraffin was removed from the unstained sections and autoradiographs were prepared by covering them with Kodak AR-10 stripping film and at varying intervals of exposure from 3 to 7 weeks, the emulsions were developed and the tissues stained with Nuclear Fast Red and Alcian Blue.

### RESULTS

The results obtained from the scintillation counter are shown in Tables I–IV. It was apparent that the distribution of the Lissamine Green V corresponded with the histological appearance of the tumour in that, in areas stained green, the tumour appeared to be viable whereas in those not reached by the dye, there was evidence of necrosis (Fig. 2). However, although the concentration of $^{35}S$ mustard and $^{3}H$ thymidine was higher in the green-stained area of the tumour, penetration still occurred in the non-stained regions.

Further, it was noticed that the maximum concentration of isotope labelled agent following injection was achieved more rapidly in the green-stained, than in

TABLE I.—$^{35}S$ *Sulphur Mustard—Scintillation Counts/Minute/Mg. of Tissue in Viable Zone*

| Time interval (min.) | Tumour number | At tumour edge A | B | At junctional zone mm. from tumour edge 4 | 5 | 6 | 7 | 5 | 3 |
|---|---|---|---|---|---|---|---|---|---|
| 15 | 1 | 933 | — | — | 892 | — | — | 841 | — |
| | 2 | 874 | 922 | — | 818 | — | — | — | — |
| | 3 | 942 | — | — | 901 | — | — | — | 918 |
| 30 | 4 | 952 | — | 898 | — | — | — | 936 | — |
| | 5 | 978 | 942 | — | 934 | — | — | — | — |
| | 6 | 994 | 820 | — | — | 925 | — | — | — |
| 45 | 7 | 1025 | 1080 | — | 1001 | — | — | — | — |
| | 8 | 998* | — | — | 977* | — | — | — | 1008* |
| | 9 | 1036* | 1116* | — | 1007* | — | — | — | — |
| 80 | 10 | 1119 | 974 | — | 1102 | — | — | — | — |
| | 11 | 1092 | 892 | — | 1111 | — | — | — | 958 |
| | 12 | 1139* | 991* | — | 1131* | — | — | 1067* | — |
| 120 | 13 | 1172 | 1264 | — | 1239 | — | — | — | — |
| | 14 | 1179† | 1100† | 1112† | — | — | — | — | — |
| | 15 | 998† | — | 1153† | — | — | — | — | — |
| 180 | 16 | 1064† | 947† | — | 940† | — | — | — | — |
| | 17 | 1184† | 1260† | — | — | — | 1284† | — | — |
| | 18 | 1103† | — | — | — | — | 1166† | — | — |

Standard 10 mμCi. = 13,771 counts. Background = 192 counts.
* Standard 10 mμCi. = 13,952 counts. Background = 271 counts.
† Standard 10 mμCi. = 13,860 counts. Background = 185 counts.

TABLE II.—$^{35}S$ *Sulphur Mustard—Scintillation Counts/Minute/Mg. of Tissue in Necrotic Zone*

| Time interval (min.) | Tumour number | mm. from junction with viable tumour 2 | 3 | 5 | 6 | 11 | 15 | 16 | 9 | 5 | 3 | 2 |
|---|---|---|---|---|---|---|---|---|---|---|---|---|
| 15 | 1 | 198 | — | — | 141 | 78 | — | — | — | 108 | — | — |
| | 2 | 164 | — | — | 116 | 64 | — | — | — | 169 | 207 | — |
| | 3 | 188 | — | — | 92 | 84 | — | — | — | 123 | — | 177 |
| 30 | 4 | — | — | 184 | — | 90 | — | — | — | — | — | 303 |
| | 5 | — | — | 171 | — | 138 | — | — | — | — | — | 347 |
| | 6 | — | 263 | — | — | 110 | — | — | — | 192 | — | — |
| 45 | 7 | 408 | — | — | 314 | 194 | — | — | — | — | — | 387 |
| | 8 | — | — | 298* | — | 181* | 153* | — | 243* | 341* | — | 369* |
| | 9 | — | — | 364* | — | 188* | 173* | — | 191* | 376* | — | — |
| 80 | 10 | 680 | — | — | — | 280 | — | — | — | — | 641 | — |
| | 11 | 672 | — | — | — | 254 | — | — | — | — | 583 | — |
| | 12 | — | — | 536* | — | 268* | — | 123* | 298* | — | 613* | — |
| 120 | 13 | — | 703 | — | — | 375 | — | — | — | 612 | — | — |
| | 14 | — | 695† | — | — | 420† | — | — | — | 627† | — | — |
| | 15 | — | 678† | — | — | 369† | — | — | — | 539† | — | — |
| 180 | 16 | — | 698† | — | — | 576† | 408† | — | 595† | — | — | 730† |
| | 17 | 713† | — | 573† | — | 480† | — | — | — | 531† | — | — |
| | 18 | 696† | — | — | 599† | 638† | — | — | — | — | 688† | — |

Standard 10 mμCi. = 13,771 counts. Background = 192 counts.
* Standard 10 mμCi. = 13,952 counts. Background = 271 counts.
† Standard 10 mμCi. = 13,860 counts. Background = 185 counts.

TABLE III.—*Thymidine-6-T(n)—Scintillation Counts/Minute/Mg. of Tissue in Viable Zone*

| Time interval (min.) | Tumour number | At tumour edge | | At junctional zone mm. from tumour edge | | | | |
|---|---|---|---|---|---|---|---|---|
| | | A | B | 4 | 5 | 7 | 5 | 3 |
| 30 | 1 | 937 | 921 | — | 941 | — | 899 | — |
| | 2 | 928 | 802 | — | 912 | — | — | 875 |
| | 3 | 891 | 936 | — | 836 | — | — | 912 |
| 60 | 4 | 942 | 958 | — | — | — | 961 | — |
| | 5 | 976 | 957 | — | — | 932 | — | 960 |
| | 6 | 953 | 926 | — | — | — | 944 | — |
| 100 | 7 | 1179† | 995† | — | 962† | — | 947† | — |
| | 8 | 981† | 1085† | — | 1022† | — | 1016† | — |
| 130 | 9 | 1172* | 1322* | 1196* | — | — | 1231* | — |
| | 10 | 1191* | 1200* | 1244* | — | — | — | 1111* |
| 180 | 11 | 1094* | 1163* | — | 1088* | — | 1039* | — |
| | 12 | 1133* | 1109* | — | 1107* | — | 1115* | — |

Standard 10 mμCi. = 13,920 counts. Background = 283 counts.
* Standard 10 mμCi. = 14,435 counts. Background = 179 counts.
† Standard 10 mμCi. = 13,541 counts. Background = 208 counts.

TABLE IV.—*Thymidine-6-T(n)—Scintillation Counts/Minute/Mg. of Tissue in Necrotic Zone*

| Time interval (min.) | Tumour number | mm. from junction with viable tumour | | | | | | | | | | |
|---|---|---|---|---|---|---|---|---|---|---|---|---|
| | | 2 | 3 | 5 | 9 | 10 | 11 | 15 | 10 | 5 | 3 | 2 |
| 30 | 1 | 415 | — | 343 | — | — | 184 | — | — | — | — | 324 |
| | 2 | — | 407 | — | — | — | 102 | — | — | 326 | — | 351 |
| | 3 | 397 | — | 339 | — | 250 | — | — | — | 411 | — | 406 |
| 60 | 4 | 571 | — | — | — | 227 | — | — | — | — | — | 525 |
| | 5 | 396 | — | — | — | — | 281 | — | — | 389 | — | — |
| | 6 | — | 483 | — | — | 296 | — | — | — | — | 406 | — |
| 100 | 7 | 683† | — | 593† | — | — | 469† | — | — | — | — | 633† |
| | 8 | 731† | — | 621† | — | 473† | — | 384† | 399† | — | — | 713† |
| 130 | 9 | 876* | — | 792* | — | — | 606* | — | — | 784* | — | 915* |
| | 10 | 861* | — | — | 678* | — | — | — | — | — | 829* | — |
| 180 | 11 | 933* | — | 902* | — | — | 762* | 619* | — | — | — | 956* |
| | 12 | — | 928* | — | — | 830* | — | 841* | 778* | 888* | — | 912* |

Standard 10 mμCi. = 13,920 counts. Background = 283 counts.
* Standard 10 mμCi. = 14,435 counts. Background = 179 counts.
† Standard 10 mμCi. = 13,541 counts. Background = 208 counts.

the non-stained areas. With both labelled agents, 90 % of maximum concentration was achieved in 1 hour in the stained area of the tumour, whereas in the non-stained portion the concentration of agent continued to rise as the time the tumour was exposed to the agent was increased (Fig. 3). Consequently, as the time interval between injection of the agent and the death of the animal increased, the difference in concentration of agent in stained and unstained areas became less (Fig. 3, 4).

A fall in concentration of agent was observed as the distance between the area sampled and the edge of the tumour increased. This fall in concentration was maximum across the first few millimetres of unstained tumour (Fig. 4). A gradient across the stained portion of tumour only occurred when the time of tumour exposure to agent was short, i.e. less than 15 minutes.

FIG. 3.—The difference in concentration of $^{35}S$ mustard in viable and necrotic areas of tumour. The readings in the viable region were taken from the tumour edge and in the necrotic zone from a point 1 cm. inside the junction with viable tumour. The concentration of labelled agent increases as the time interval between its injection and the death of the animal becomes greater.

FIG. 4.—The fall in concentration of isotope labelled agent is maximum across the first 2 mm. of unstained tumour. 100% concentration represents the highest level of agent found in the stained peripheral portion of the tumour.

Samples of skin and muscle contained isotope-labelled agent in a concentration of 20% less than that obtained at the periphery of the stained tumour areas. Whether or not these findings support the concept of selective tumour uptake has not been established, since samples from other tissues and organs such as liver and kidney, which might have shown a higher level of agent, were not studied.

In support of observations recorded previously, namely, the presence of isotope-labelled agent in unstained areas of tumour, uptake of $^{3}$H thymidine was demonstrated in these areas by means of an autoradiographic technique. Although the unstained regions were largely necrotic, islands of cells with a histologically viable appearance occurred in them and further, these cells were seen to take up $^{3}$H thymidine (Fig. 5–8). This established the viability of these cells since thymidine is only taken up by cells actively synthesising deoxyribonucleic acid (DNA) (Reichard and Estborn, 1951; Friedkin *et al.*, 1956; Hughes *et al.*, 1958; Kisieleski *et al.*, 1961).

### DISCUSSION

In contrast to the distribution of the dye, labelled cytotoxic agent was found in all areas of the tumour examined and it would appear that the distribution of any agent administered will depend, to some extent, upon such physical characteristics as molecular weight, structure and electrical charge. The superior penetration of $^{35}$S mustard as compared with Lissamine Green V may be related to their molecular weights of 170 and 576 respectively. Of course some low molecular weight substances become rapidly bound to protein and might then behave more like Lissamine Green V.

In the unstained, largely necrotic regions of the tumour, drug concentration begins to reach levels comparable with those reached in the stained viable areas only when exposure to the drug has continued for a long time, e.g. 25% of the concentration present in the viable region is achieved only after 80 minutes' exposure (Fig. 4). When this time of exposure is short, i.e. less than 15 minutes, penetration of agent corresponds closely with that of the dye. Maximum concentration of agent is achieved more rapidly in the viable areas of tumour, e.g. 90% of maximum concentration being achieved in 1 hour (cf. Rubini *et al.*, 1960). These findings support the work of Shapiro and Landing (1948) who found the distribution of flourescein in sarcoma 180 in mice to be determined by the length of time after injection and the presence or absence of necrosis in the tumour.

Moving from the periphery towards the centre of the tumour, the concentration of agent becomes less and a rapid fall occurs across the first few millimetres of the unstained necrotic area. It must be remembered that the physical conditions

---

### EXPLANATION OF PLATES

FIG. 2.—A 12 day old Walker 256 tumour (3·5 cm. diameter) following the intravenous injection of Lissamine Green V. The unstained central area of tumour is largely necrotic.

FIG. 5.—Autoradiograph using thymidine, from the centre of tumour. Small island of viable cells labelled with thymidine within predominantly necrotic tissue. ×225.

FIG. 6.—Enlarged portion of Fig. 5. This area of tumour is unstained by the dye although some cells take up thymidine as shown by the black granules. ×500.

FIG. 7.—Autoradiograph taken from the periphery of the same tumour as shown in Fig. 5. This is a fully viable area which is readily penetrated by Lissamine Green V injected intravenously. ×225.

FIG. 8.—Higher magnification of Fig. 7 with a large number of heavily labelled cells. ×500.

2

5

7

6

8

inside the necrotic centre of this transplanted tumour which becomes vascularised with some difficulty, may not be the same as occur in the necrotic areas of spontaneous tumours. If the same situation does exist however, then the penetration of cytotoxic agent will be limited in a large tumour, especially when areas of necrosis are present. That penetration of cytotoxic drugs into these regions is important is shown by the ability of cells present in these areas to take up thymidine and therefore to synthesise DNA. When treatment of human tumours is broken off because of general toxicity, some of the surviving tumour cells may divide and provide a sufficient " background growth " ultimately to produce therapeutic failure, without the need to invoke any mechanism of resistance (Klein, 1961).

These results indicate that, in the Walker 265 tumour, penetration of cytotoxic agent is a function of tumour mass, the presence or absence of necrosis, the time of exposure to the drug and the physical characteristics of the agent used. Initial observations, which have since been made in eight different human tumours, appear to confirm the importance of these factors in determining the penetration of cytotoxic drugs and the accessibility of many cancer cells to cytotoxic drugs in large malignant tumours especially those with considerable areas of necrosis, will be limited. This will apply particularly to many alkylating agents with a short biological half-life, chosen because of their ability to produce less side effects and which, therefore, come into contact with the tumour for only a short time while in an active form. It is possible also that an antimetabolite, having a specific affinity for a particular molecule present in the viable part of a tumour but not in a largely necrotic area (e.g. methotrexate and dihydrofolate reductase), might be diverted from the latter by mass-action. However, if the molecular structure remains simple, the use of an antimetabolite which persists for a long time and would ultimately reach the necrotic part of the tumour may be indicated in preference to a rapidly inactivated alkylating agent in the treatment of a large tumour mass.

The results obtained in this study support the statement by Larionov (1959) based on clinical material that " the degree of anti tumour effect is inversely proportional to the mass of tumour tissue ".

SUMMARY

1. The distribution of $^{35}S$ mustard and $^{3}H$ thymidine was examined in 30 Walker 256 carcino-sarcomata.

2. Comparisons between the penetration of these agents and the dye Lissamine Green V were made.

3. Penetration of agent was found to be a function of tumour mass, the presence or absence of necrosis, the time of exposure to the drug and the physical characteristics of the agent used.

4. The possible relevance of these findings to the use of cytotoxic agents in the treatment of malignant human tumours was discussed.

This work was carried out at the Royal Marsden and Westminster Hospitals, London. I am indebted to Mr. J. D. Griffiths who first stimulated my interest in this subject and to Professor H. Ellis and Dr. L. Cobb for advice and Miss Rosemary Ellis for technical assistance. Part of this work was supported by a grant from the Board of Governors' Discretionary Fund, Westminster Hospital.

### REFERENCES

ALGIRE, G. H. AND CHALKLEY, H. W.—(1945) *J. natn. Cancer Inst.*, **6**, 73.
ALGIRE, G. H.—(1947) *A.A.A.S. Symposium.* ' Approaches to Tumour Chemotherapy, ' Maryland, 1945–6. Pennsylvania (Science Press Printing Co.), p. 13.
BRAITHWAITE, J. L.—(1958) *Br. J. Cancer*, **12**, 75.
FRIEDKIN, M., TILSON, D. AND ROBERTS, D. W.—(1956) *J. biol. Chem.*, **220**, 627.
GOLDACRE, R. J. AND SYLVÉN, B.—(1962) *Br. J. Cancer*, **16**, 306.
GOLDMANN, E. E.—(1911) *Beitr. klin. Chir.*, **72**, 1.
HUGHES, W. L., BOND, V. P., BRECHER, G., CRONKITE, E. P., PAINTER, R. B., QUASTLER, H. AND SHERMAN, F. G.—(1958) *Proc. natn. Acad. Sci., U.S.A.*, **44**, 476.
IDE, G. A., BAKER, N. H. AND WARREN, S. L.—(1939) *Am. J. Roentg.*, **42**, 891.
KISIELESKI, W. E., BASERGA, R. AND LISCO, H.—(1961) *Atomphaxis*, **7**, 81.
KLEIN, G.—(1961) ' Biological Approaches to Cancer Chemotherapy ', Edited by R. J. C. Harris. London & New York (Academic Press) p. 210.
LARIONOV, L. F.—(1959) *Acta Un. int. Cancr.*, **15**, 42.
LEWIS, W. H.—(1927) *Johns Hopkins Hosp. Bull.*, **41**, 156.
NEWTON, K. A.—(1965) *Br. J. Radiol.*, **38**, 224.
REICHARD, P. AND ESTBORN, B.—(1951) *J. biol. Chem.*, **188**, 839.
RUBINI, J. R., CRONKITE, E. P., BOND, V. P., AND FLIEDNER, T. M.—(1960)., *J. clin. Invest.*, **39**, 909.
SAITO, M.—(1937) *J. Jap. surg. Soc.*, **31**, 372.
SAMPSON, J. A.—(1912) *Surgery Gynec. Obstet.*, **14**, 215.
SHAPIRO, D. M. AND LANDING, B. H.—(1948) *Science, N.Y.*, **108**, 304.
SHINKAWA, T.—(1939) *Nagoya J. med. Sci.*, **13**, 263.
SMART, P. J. G., MCKINNA, J. A. AND GRIFFITHS, J. D.—(1963) *Br. J. Surg.*, **50**, 766.
WATERS, H. G. AND GREEN, J. A.—(1959) *Cancer Res.*, **19**, 326.